# The Rab5 Effector Rabankyrin-5 Regulates and Coordinates Different Endocytic Mechanisms

**DOI:** 10.1371/journal.pbio.0020261

**Published:** 2004-08-24

**Authors:** Carsten Schnatwinkel, Savvas Christoforidis, Margaret R Lindsay, Sandrine Uttenweiler-Joseph, Matthias Wilm, Robert G Parton, Marino Zerial

**Affiliations:** **1**Max-Planck Institute of Molecular Cell Biology and GeneticsDresdenGermany; **2**Laboratory of Biological Chemistry, Medical SchoolUniversity of Ioannina, IoanninaGreece; **3**Institute for Molecular Bioscience, Centre for Microscopy and MicroanalysisSchool of Biomedical Sciences, University of Queensland, Brisbane, QueenslandAustralia; **4**European Molecular Biology LaboratoryHeidelbergGermany

## Abstract

The small GTPase Rab5 is a key regulator of clathrin-mediated endocytosis. On early endosomes, within a spatially restricted domain enriched in phosphatydilinositol-3-phosphate [PI(3)P], Rab5 coordinates a complex network of effectors that functionally cooperate in membrane tethering, fusion, and organelle motility. Here we discovered a novel PI(3)P-binding Rab5 effector, Rabankyrin-5, which localises to early endosomes and stimulates their fusion activity*.* In addition to early endosomes, however, Rabankyrin-5 localises to large vacuolar structures that correspond to macropinosomes in epithelial cells and fibroblasts. Overexpression of Rabankyrin-5 increases the number of macropinosomes and stimulates fluid-phase uptake, whereas its downregulation inhibits these processes. In polarised epithelial cells, this function is primarily restricted to the apical membrane. Rabankyrin-5 localises to large pinocytic structures underneath the apical surface of kidney proximal tubule cells, and its overexpression in polarised Madin-Darby canine kidney cells stimulates apical but not basolateral, non-clathrin-mediated pinocytosis. In demonstrating a regulatory role in endosome fusion and (macro)pinocytosis, our studies suggest that Rab5 regulates and coordinates different endocytic mechanisms through its effector Rabankyrin-5. Furthermore, its active role in apical pinocytosis in epithelial cells suggests an important function of Rabankyrin-5 in the physiology of polarised cells.

## Abstract 2

Rabankyrin-5, which is activated by the small GTPase Rab5, coordinates two disparate methods that cells use to take in solids and fluids, playing a key role in both endosome fusion and macropinocytosis

## Introduction

In mammalian cells multiple mechanisms of endocytosis operate within a single cell to perform nutrient uptake, cellular homeostasis, neurotransmission, signal transduction, antigen presentation, and defence against pathogens. Endocytosis comprises two major categories, phagocytosis and pinocytosis, depending on the uptake of particles or fluid, respectively (reviewed in Conner and Schmid 2003). Pinocytosis encompasses various membrane entry routes and mechanisms. Clathrin-mediated endocytosis, the best-studied route at the molecular level to date, primarily serves receptor-mediated uptake. Caveolae assemble on sphingolipid and cholesterol rafts and may internalise molecules partitioning in these lipid microdomains. To a certain extent, ligand-receptor complexes and raft components can follow both entry routes but can also be internalised via clathrin- and caveolae-independent endocytosis. Large volumes of fluid are engulfed by the closure of plasma membrane protrusions in a process termed macropinocytosis. Although it was the first mode of pinocytosis reported (Lewis 1931), little is known on the molecular mechanisms underlying this physiologically very important endocytic process. Constitutively active in immature dendritic cells, macropinocytosis favors antigen sampling (Steinman and Swanson 1995). However, it can be transiently induced in most cells by growth factors (Haigler et al. 1979; Shao et al. 2002), tumor-promoting chemicals such as phorbol 12-myristate 13-acetate (PMA), or oncogenes like H-Ras or v-Src (Bar-Sagi and Feramisco 1986; Veithen et al. 1996), and it has been proposed to downregulate signalling molecules from the cell surface. Since macropinosomes arise from membrane ruffles, regions of intense actin remodelling which are also a trait of motile cells, they have been implicated in directed cell locomotion (Carpentier et al. 1991). Macropinocytosis is exploited by several invasive pathogens as entry route (Francis et al. 1993; Sansonetti 1997) but differs from phagocytosis with respect to regulation (e.g., receptor mediated) and cargo (e.g., uptake and degradation of opsonised particles) (Galan and Zhou 2000). Macropinosomes are distinct from early endosomes, morphologically and biochemically. In epidermal growth factor (EGF)-stimulated A431 cells, whereas macropinosomes can fuse homotypically, they seldom fuse with early endosomes (Hewlett et al. 1994). However, this partition is not absolute, since in dendritic cells macropinosomes do fuse with endosomes (Racoosin and Swanson 1993). The intracellular trafficking properties of macropinosomes may therefore be governed by cell type-specific mechanisms and fulfil specialised functions. Finally, macropinocytosis shares mechanistic features with apical fluid-phase endocytosis in polarised cells (Gottlieb et al. 1993; Jackman et al. 1994; Holm et al. 1995; Amyere et al. 2002). In kidney, apical pinocytosis contributes an essential function to the physiology of the renal system by contributing to vectorial fluid-phase transport across the membranes (Goligorsky and Hruska 1986).

Given the dependence on actin remodelling, it is no surprise that Rho GTPases (West et al. 2000), ARF6 (Radhakrishna et al. 1996), and type 1 phosphatidylinositol-3 kinases (PI3-Ks) (Hooshmand-Rad et al. 1997) are involved in macropinocytosis, presumably through their role in membrane ruffling. Much less clear is the function of two Rab GTPases, Rab5 and Rab34/Rah, both of which are implicated in the formation of macropinosomes (Li et al. 1997; Sun et al. 2003). Rab34/Rah colocalises with actin to membrane ruffles and nascent macropinosomes and its overexpression promotes macropinocytosis (Sun et al. 2003). Rab5 regulates fluid-phase and receptor-mediated uptake (Bucci et al. 1992; McLauchlan et al. 1998), and its activity has been linked to Ras-stimulated fluid-phase endocytosis (Li et al. 1997). However, Rab5 colocalises to some, but not all, macropinosomes harbouring Rab34/Rah (Sun et al. 2003). Furthermore, Rab5 together with its effector PI3-K, hVPS34, regulates the recruitment of a set of cytosolic FYVE-finger effector proteins on early endosomes. These molecules cooperate in the tethering and fusion of clathrin-coated vesicles (CCVs) with early endosomes and homotypic endosome fusion (Gorvel et al. 1991; Bucci et al. 1995; Rubino et al. 2000) as well as the motility of early endosomes along microtubules (Nielsen et al. 1999). However, in view of the function of Rab5 on the early endosomes, its role in macropinocytosis is difficult to assess. Specifically, it is unclear whether the large vacuoles induced by Rab5 activation, either via the expression of activated mutants (Stenmark et al. 1994) or via signalling molecules that stimulate the GTP loading on Rab5 (Lanzetti et al. 2000; Tall et al. 2001), correspond to either macropinosomes or enlarged endosomes originated by increased homotypic early-endosome fusion, or both (Hewlett et al. 1994). Here, the identification of a novel Rab5 FYVE-finger effector, Rabankyrin-5, led us to revisit the role of Rab5 in fluid-phase (macro)pinocytosis in both nonpolarised and polarised cells.

## Results

### Identification of a Novel 130-kDa Rab5 Effector

**Figure 1:**
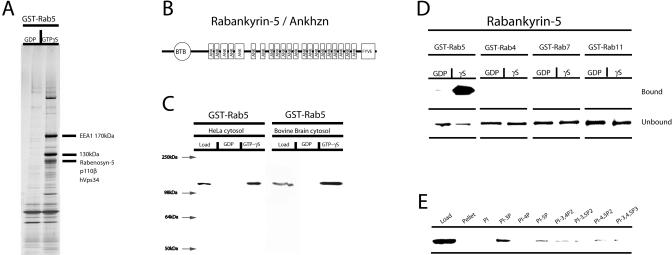
A Protein of 130 kDa Is a New Rab5 Effector (A) GST-Rab5-GDP and GST-Rab-GTPγS were loaded on beads and incubated with bovine brain cytosol. Bound proteins were eluted and analysed by SDS-PAGE followed by Coommasie Blue staining. The positions of the already known Rab5 effectors (EEA1, Rabaptin-5, hVps34, p110β, and Rabenosyn-5) and of the new Rab5 effector are indicated. (B) Schematic representation of the domain organisation in Rabankyrin-5. ANK, ankyrin repeats. (C) Bovine brain cytosol or HeLa cell cytosol was incubated with GST-Rab5-GDP– or GST-Rab5-GTPγS–loaded beads. Subsequently the beads were washed, and bound proteins were eluted and analysed by Western blotting using anti–Rabankyrin-5 antibodies. (D) GST-Rab4, -5, -7, and -11 fusion proteins were preloaded with GDP or GTPγS and incubated with in vitro-translated ^35^S-methionine–labelled Rabankyrin-5 full-length protein. As a control, bound and unbound material was analysed by SDS-PAGE followed by phosphoimager analysis. (E) Rabankyrin-5 binds most strongly to PI(3)P. Recombinant full-length Rabankyrin-5 was incubated with liposomes containing 2% of the indicated phosphoinositide. Bound Rabankyrin-5 was detected by Western blotting.

We have previously purified a large number of Rab5 effectors by an affinity chromatography approach based on glutathione S-transferase (GST)-Rab5-GTPγS (Christoforidis et al.1999a). Among the numerous proteins purified, we focused on a prominent 130-kDa protein (Figure 1A). The protein was digested with trypsin and the amino acid sequences of the tryptic fragments were determined by nanoelectrospray tandem mass spectrometry (Wilm et al. 1996). The identified peptides matched the mouse sequence of Ankhzn (unpublished data). The corresponding human cDNA was obtained by PCR, using primers derived from the mouse DNA sequence and a random primed HeLa cDNA library as template (Zerial et al. 2001). The sequence of the human cDNA matched the recently revised hAnkhzn gene sequence (NP 057460.2) (Kuriyama et al. 2000). The predicted human p130 primary sequence consists of an N-terminal BTB/POZ domain, a C-terminal FYVE-finger, and 21 successive ankyrin (ANK) repeats in between these two domains (Figure 1B). In light of the fact that p130 is a Rab5 effector (this study), we propose to amend its name to Rabankyrin-5 (**Rab5** binding and **ankyrin** repeats containing protein).

To investigate the function of human Rabankyrin-5, we raised antibodies against the recombinant full-length protein. By Western blotting, these antibodies detected a predominant band of 130 kDa in both bovine brain and HeLa cytosol, in the corresponding GST-Rab5-GTPγS but not GST-Rab5-GDP affinity column eluates (Figure 1C). Evidence for a direct interaction with Rab5 was obtained by incubating in vitro-translated Rabankyrin-5 with beads displaying GST-Rab5, -Rab4, -Rab7, and -Rab11, preloaded with either GDP or GTPγS. Figure 1D shows that human Rabankyrin-5 binds to GST-Rab5-GTPγS, but neither to beads containing GST-Rab5-GDP nor to other Rab proteins. We conclude that Rabankyrin-5 binds to Rab5 specifically, directly, and GTP dependently.

### Association of Rabankyrin-5 with Two Types of Rab5-Positive Vesicles

**Figure 3:**
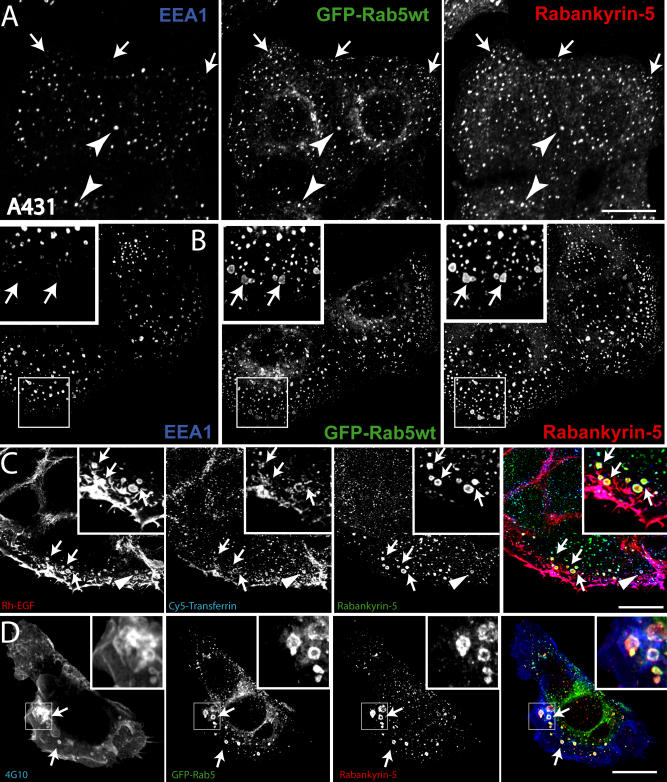
Rabankyrin-5 Associates with Two Types of Rab5-Containing Vesicles in A431 Cells, Early Endosomes, and Macropinosomes (A) A431 cells stably transfected for GFP-Rab5wt were immunostained for endogenous Rabankyrin-5 and EEA1. While there is perinuclear overlap between Rab5, Rabankyrin-5, and EEA1 in nontransfected cells (arrowheads), some smaller peripheral structures are devoid of EEA1 (arrows). (B) Overexpression of Rabankyrin-5 in A431 by using a recombinant adenovirus construct of Rabankyrin-5 causes an accumulation of peripheral, enlarged Rab5-positive structures, costained mainly by Rabankyrin-5 (arrows) but not detectable for EEA1. (C) Rabankyrin-5 localises on EGF-induced and -enriched macropinosomes. Serum-starved A431 cells (16 h) were incubated for 7 min with 100 ng/ml rhodamine-conjugated EGF to induce macropinocytosis and 1 μg/ml Cy5-labelled transferrin. Endogenous Rabankyrin-5 localises to enlarged EGF-containing macropinosomes, indicated by the lack of transferrin labelling (arrows), but also to EGF- and transferrin-containing endosomes (arrowheads). (D) Rabankyrin-5 structures contain tyrosine-phosphorylated proteins. A431 cells, stably transfected for GFP-Rab5, were stimulated with 50 ng/ml EGF for 7 min and immediately processed for immunofluorescence. Costaining of Rabankyrin-5 and tyrosine-phosphorylated proteins (α-4G10) reveal the localisation of Rabankyrin-5 to plasma membrane ruffles. Scale bars represent 10 μm.

After EEA1 (Stenmark et al. 1996) and Rabenosyn-5 (Nielsen et al. 2000), Rabankyrin-5 is the third Rab5 effector containing a FYVE-finger. Since the FYVE-finger is a key determinant for targeting these proteins to early endosomes via its binding to phosphatidylinositol-3-phosphate [PI(3)P] (Kutateladze et al. 1999), we tested whether this holds true also for Rabankyrin-5. First, recombinant Rabankyrin-5 interacted significantly with PI(3)P in a liposome-binding assay (Figure 1E). Second, endogenous Rabankyrin-5 colocalised significantly with EEA1 and Rab5-positive early endosomes (approximately 80%) in A431 cells by confocal immunofluorescence microscopy analysis (see Figure 3A and 3B below).

**Figure 2:**
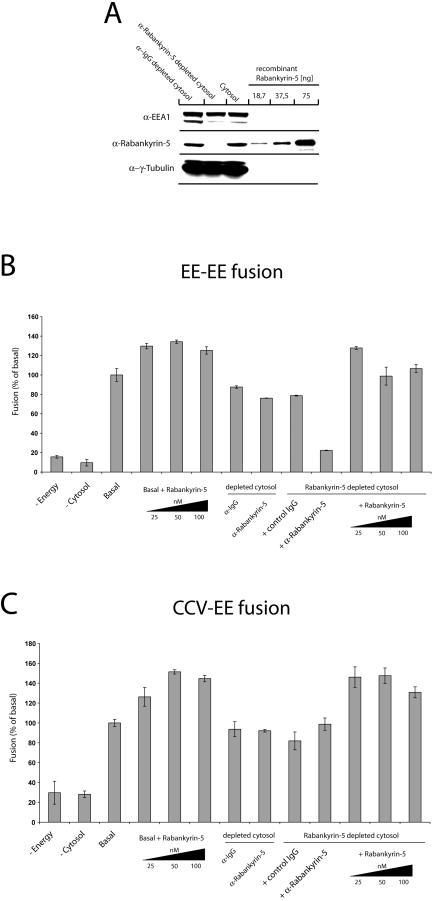
Rabankyrin-5 Stimulates Homotypic Fusion between Early Endosomes and Heterotypic Fusion between Early Endosomes and CCVs In Vitro (A) Rabankyrin-5 was efficiently immunodepleted from HeLa cytosol without affecting the EEA1 concentration. Twenty micrograms of HeLa cell cytosol, either treated with control IgGs and immunodepleted for Rabankyrin-5 or nontreated, was applied to SDS-PAGE and probed for the indicated antigens by Western blotting. Anti-γ tubulin was used as a loading control. (B and C) Addition of recombinant Rabankyrin-5 stimulates homotypic and heterotypic fusion. Fusion (B) between early endosomes (EE-EE fusion) and (C) of early endosomes to CCVs (CCV-EE fusion) in vitro was performed in the presence of 3 mg/ml HeLa cytosol and an ATP-regenerating system (basal) and in the absence or presence of the indicated reagents. For some conditions, cytosol was immunodepleted for Rabankyrin-5 (columns 8–13) and in addition supplemented with anti–Rabankyrin-5 antibodies to neutralise the function of membrane-associated proteins (column 10). Background fusion activity is demonstrated by ATP (−Energy) or cytosol (−Cytosol) omission.

We next investigated whether Rabankyrin-5 plays a role in homotypic early endosome fusion as well as fusion of CCVs with early endosomes, similar to EEA1 (Christoforidis et al. 1999a) and Rabenosyn-5 (Nielsen et al. 2000). Quantitative immunodepletion of the cytosolic pool of Rabankyrin-5 using anti–Rabankyrin-5 antibodies (Figure 2A) had only minor effects on early endosome fusion in vitro compared to preimmune serum (Figure 2B). Considering the possibility that the early-endosome–associated pool of Rabankyrin-5 may still be sufficient for fusion, we attempted to neutralise its function by the addition of anti–Rabankyrin-5 antibodies on top of Rabankyrin-5–depleted cytosol. This treatment strongly inhibited early-endosome fusion (Figure 2B). The fusion of CCVs with early endosomes was minimally affected under these conditions (Figure 2C), suggesting that Rabankyrin-5 is not essential in this process, at least in vitro. However, addition of increasing amounts of recombinant Rabankyrin-5 (at concentrations in the range of the endogenous protein; see Materials and Methods) on either immunodepleted cytosol or nontreated cytosol significantly enhanced both homo- and heterotypic fusion activity. We conclude from these experiments that endogenous Rabankyrin-5 colocalises with EEA1 on Rab5 endosomes in vivo, through the interaction with Rab5 (see Figure 1D) and PI(3)P (see Figure 1E). It plays a modulatory role in early-endosome fusion since it can stimulate both homotypic and heterotypic fusion of CCVs with early endosomes. However, it does not appear to be strictly required in the latter reaction in vitro.

Surprisingly, we noted that some peripheral structures harbouring Rabankyrin-5 overlapped with Rab5 but either poorly or not at all with EEA1 in A431 cells (Figure 3A, arrows). Upon overexpression, Rabankyrin-5 induced the accumulation of several vesicles of variable size in the periphery of the cells, most of them lacking EEA1 (Figure 3B, arrows). The irregular size, intracellular distribution (Swanson 1989), and lack of endosomal markers (see “Rabankyrin-5 localises to macropinosomes” below) (Hewlett et al. 1994) of these structures were suggestive of (macro)pinocytic vesicles. We therefore set out to investigate the putative role of Rabankyrin-5 in macropinocytosis.

### Rabankyrin-5 Localises to Macropinosomes

Macropinosomes have been so far defined by a combination of criteria rather than specific molecular markers. To test whether the Rabankyrin-5 vesicles fulfil the previously described requirements of macropinosomes, we determined whether they (1) are stimulated by growth factors (Haigler et al. 1979; West et al. 1989; Amyere et al. 2000), (2) do not promote receptor-mediated endocytosis of transferrin, (3) engulf large amounts of extracellular fluid (Hewlett et al. 1994; Amyere et al. 2000), (4) depend on PI3-K activity (Araki et al. 1996; Zhou et al. 1998), and (5) follow actin rearrangements at the plasma membrane. A431 and NIH3T3 cells are established experimental systems that exhibit different features in the regulation of macropinocytosis. In A431 cells, macropinocytosis can be transiently induced upon growth factor stimulation (Hewlett et al. 1994), whereas NIH3T3 cells exhibit constitutive macropinocytic activity (Dharmawardhane et al. 2000). We first tested whether EGF can induce the formation of Rabankyrin-5–positive macropinocytic structures. Upon treatment of A431 cells with rhodamine-conjugated EGF, endogenous Rabankyrin-5 colocalised to enlarged structures which, importantly, were largely devoid of simultaneously internalised transferrin (Figure 3C). Interestingly, EGF was not excluded but rather enriched in these structures. In addition, we observed that such vesicles were also enriched in tyrosine-phosphorylated proteins, presumably EGF-receptor and downstream signalling molecules (Figure 3D).

**Figure 4:**
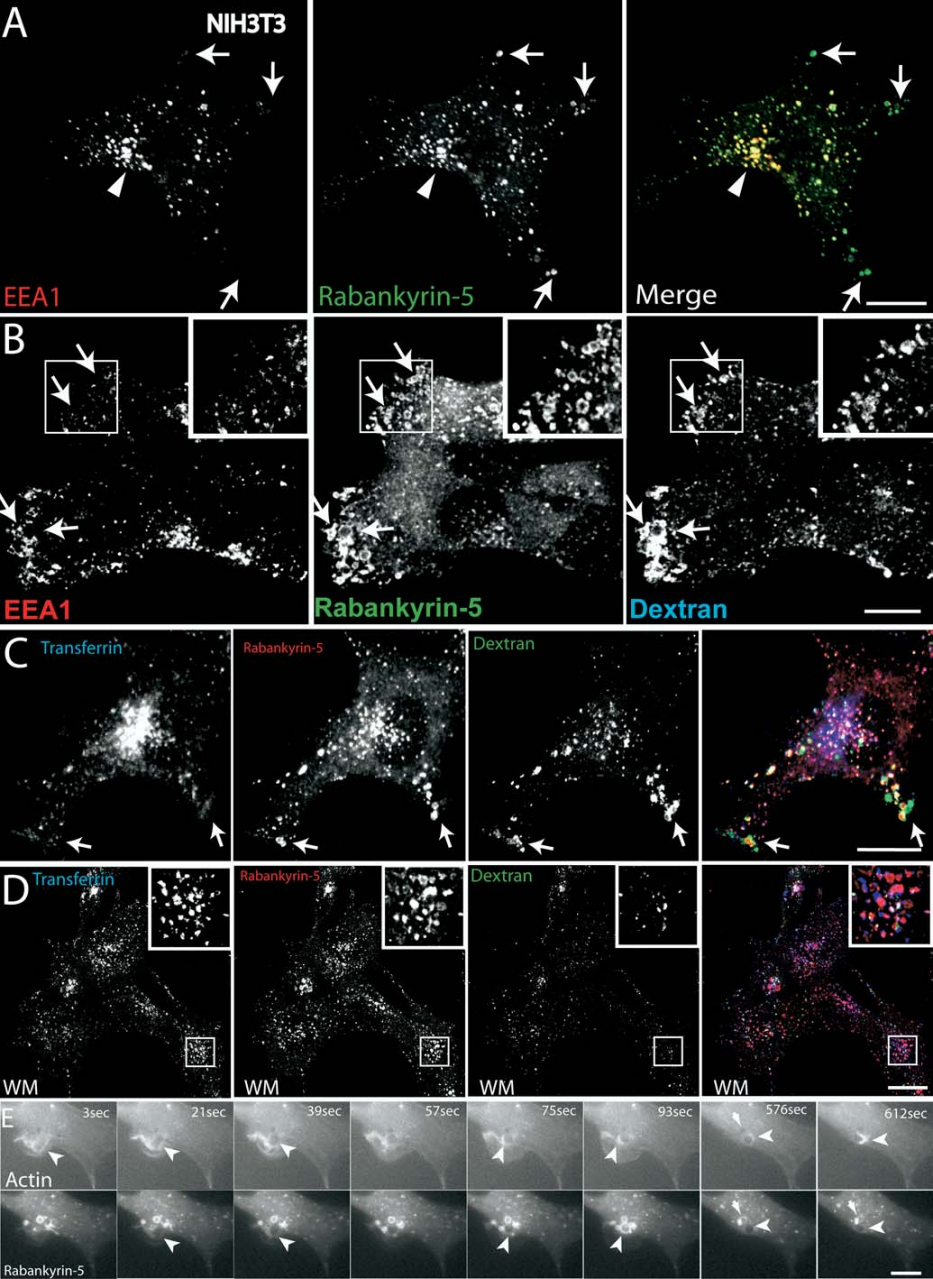
Rabankyrin-5 Localises to Macropinosomes in NIH3T3 Cells (A) NIH3T3 cells directly fixed and immunostained for endogenous Rabankyrin-5 and EEA1 reveal segregation of Rabankyrin-5 from EEA1-containing structures (arrows) in the cell periphery, while there is colocalisation in the cell centre (arrowheads). (B) Overexpression of Rabankyrin-5 increases the number of peripheral enlarged structures devoid of EEA1. NIH3T3 cells were infected with recombinant adenovirus for Rabankyrin-5 for 18 h. Dextran (2,5 mg/ml) uptake was performed for 3 min at 37 °C, fixed, and immunostained for the indicated antigens. (C and D) Formation of enlarged Rabankyrin-5 structures requires PI3-K activity. NIH3T3 cells either (C) DMSO treated or (D) pretreated for 20 min with wortmannin (WM; 100 nM) were incubated for 8 min with 0,5 μg/ml rhodamine-labelled transferrin and 2,5 mg/ml FITC-labelled dextran (MW, 10.000), fixed, processed for immunofluorescence, and analysed by confocal scanning microscopy. (E) NIH3T3 cells transiently transfected for YFP-Rabankyrin-5 and CFP-actin were imaged using time-lapse video microscopy to visualise the formation of macropinosomes by actin-driven membrane ruffles. Images were taken for the indicated time points. The arrowhead points towards Rabankyrin-5 association to an enlarged vesicle driven by actin dynamics over time. Scale bars represent 10 μm.

**Figure 5:**
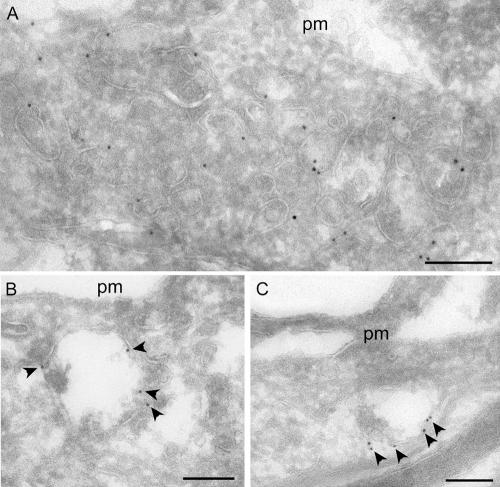
Immunolocalisation of Endogenous and Overexpressed Rabankyrin-5 in NIH3T3 Cells Untransfected NIH3T3 cells or cells transfected with Rabankyrin-5 were labelled with antibodies to Rabankyrin-5 followed by 10 nm protein A gold. (A) Transfected cell showing labelling of a group of vesicular structures underlying the plasma membrane (pm). (B and C) In control (untransfected) cells, low but specific labelling for Rabankyrin-5 (arrowheads) is associated with compartments close to the pm. Scale bars represent 200 nm.

In NIH3T3 fibroblasts, the staining pattern of endogenous Rabankyrin-5 was similar to that of EGF-stimulated A431 cells. Rabankyrin-5 was detected on enlarged structures, predominantly at the outermost periphery and in membrane protrusions of the cell (Figure 4A, arrows). Consistent with these observations, immunoelectron microscopy revealed that antibodies to Rabankyrin-5 labelled electron-lucent vesicular structures underlying the plasma membrane in the cell periphery and in cell protrusions (Figure 5). The labelled structures were sparse in untransfected cells, but in Rabankyrin-5– overexpressing cells fortuitous sections revealed concentrated areas of Rabankyrin-5–labelled structures. Unfortunately, we failed to distinguish these structures from conventional early endosomes by double immunolabelling experiments due to technical limitations. By light microscopy, the appearance of vacuole-shaped structures devoid of the early endosomal marker EEA1 was dramatically increased upon Rabankyrin-5 overexpression. In addition, after a 3-min pulse, these structures were intensively labelled by a fluid-phase marker (see Figure 4B, arrows) but not by transferrin (unpublished data). Given the potent induction of macropinosomes by Rabankyrin-5 in NIH3T3 cells, we used this experimental system to explore further the regulatory parameters of macropinocytosis.

In NIH3T3 cells, Rabankyrin-5–enlarged structures were labelled by an 8-min uptake of fluorescein isothiocyanate (FITC)-dextran (MW, 10.000). Consistent with their macropinocytic nature, these structures were largely depleted of transferrin (see Figure 4C, arrows) and susceptible to the PI3-K inhibitors wortmannin (see Figure 4D) and LY 294002 (unpublished data). These drugs both reduced the formation of peripheral enlarged vesicles and almost completely blocked the internalisation of high-molecular-weight dextran (MW, 70.000) (Figure S1A) that enters the cell almost exclusively via macropinocytosis (Dharmawardhane et al. 2000). Interestingly, we noted that, unlike EEA1, Rabankyrin-5 did not readily dissociate from membranes (see Figure 4D) upon inhibition of PI3-K activity, suggesting that endosome-targeting determinants other than PI(3)P contribute to its localisation. Neither markers of late endosomes (Rab7) (Chavrier et al. 1990), nor of caveosomes (caveolin) (Pelkmans et al. 2001), nor of the clathrin-independent pathway which is used by glycosyl-phosphatidylinositol (GPI)-anchored proteins (GFP-GPI) (Sabharanjak et al. 2002) overlapped significantly with the peripheral enlarged Rabankyrin-5 structures (unpublished data).

To confirm that the Rabankyrin-5 structures originated from the plasma membrane through an actin-driven process, we employed time-lapse video fluorescence microscopy on NIH3T3 cells transiently coexpressing yellow fluorescent protein (YFP)-Rabankyrin-5 and cyan fluorescent protein (CFP)-β-actin. In the sequence depicted in Figure 4E (see also Video S1), membrane ruffling driven by actin resulted in the formation of a large, plasma membrane-derived vesicle acquiring Rabankyrin-5. Some newly formed vesicles shed off Rabankyrin-5 over time and seemed to regurgitate back to the plasma membrane via an actin comet-tail. Altogether, the data support the hypothesis that the enlarged structures containing Rabankyrin-5 are macropinosomes and can be molecularly distinguished from early endosomes by being mostly, albeit not exclusively, devoid of EEA1. Moreover, and contrary to earlier findings (West et al. 1989), we found that in A431 cells, EGF but not transferrin could be enriched in macropinosomes.

### Both Rab5 and Rabankyrin-5 Are Required for Macropinocytosis

**Figure 6:**
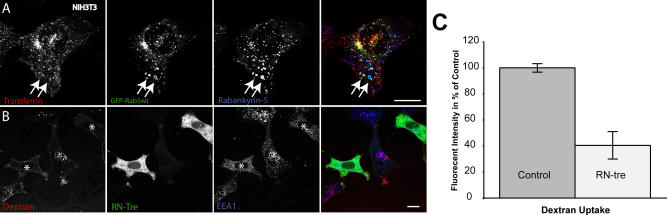
Inhibition of Rab5 Activity Decreases Fluid-Phase Uptake NIH3T3 cells transiently transfected for either (A) GFP-Rab5wt or (B) RN-tre were subjected to a 30-min uptake of rhodamine-conjugated transferrin (1 μg/ml) or dextran (MW, 70.000; 3 mg/ml) at 37 °C and further processed for confocal imaging with indicated antibodies. (A) Rab5wt transfected cells show colocalisation of Rabankyrin-5–labelled macropinocytic structures, indicated by the lack of transferrin accumulation, with Rab5. (B) Cells transiently transfected for RN-tre (asterisk) show a significant reduction of fluid-phase uptake compared to nontransfected cells. (C) Fluid-phase dextran quantification of single cells transfected for RN-tre by measuring internalised fluorescence intensity (*p* > 0,001). Values shown are means ± standard deviation of at least 15 cells. Scale bars represent 10 μm.

The GTP-dependent interaction with Rab5 suggests that Rabankyrin-5 may function as a downstream effector of Rab5 in the macropinocytic pathway. Accordingly, one would expect that Rab5 localises to macropinosomes and, together with Rabankyrin-5, is required for macropinocytosis. We found that Rab5 indeed colocalises with Rabankyrin-5 on macropinosomes (see Figure 3B, 3D, and 6A; unpublished data), which are operationally defined as vesicles (1) enriched in fluid-phase marker but depleted of transferrin receptor (Racoosin and Swanson 1992), (2) of large size (0,2–2 μm) (Swanson 1989; Hewlett et al. 1994), and (3) negative for EEA1 but positive for Rabankyrin-5. Consistent with a requirement for Rab5 in macropinocytosis, overexpression of RN-tre, a GTPase-activating protein (GAP) for Rab5 (Lanzetti et al. 2000), significantly reduced (by 60% ± 10%) the uptake of large fluorescent dextran (MW, 70.000) into transferrin- and EEA1-negative structures in NIH3T3 cells (Figure 6B and 6C). Similar results were also obtained when NIH3T3 cells were cotransfected for Rab5S34N and Rabankyrin-5 (unpublished data), implying that Rabankyrin-5 induces macropinocytosis Rab5-GTP dependently.

**Figure 7:**
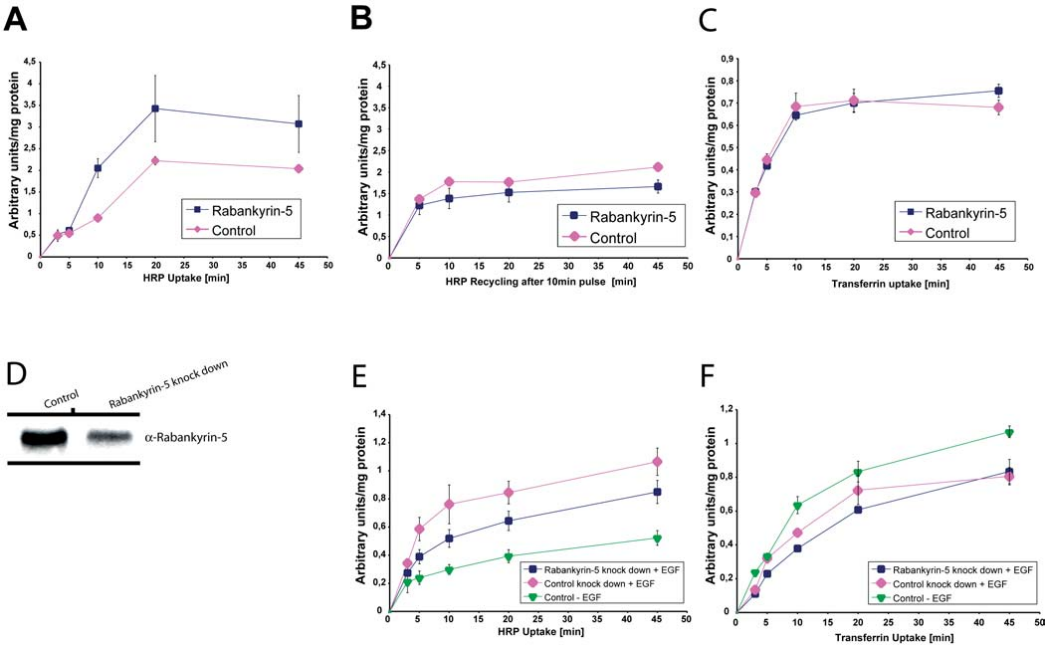
Rabankyrin-5 Overexpression Increases, whereas Knock-Down Decreases Fluid-Phase Uptake, Specifically (A–C) NIH3T3 cells were either mock-infected or infected with recombinant adenovirus encoding Rabankyrin-5. Simultaneous uptake of HRP (5 mg/ml) and biotinylated transferrin (2 μg/ml) was performed at 37 °C for the indicated time points. (C) NIH3T3 cells were pulsed with HRP (10 mg/ml) for 10 min. Recycled HRP into the medium was determined after the indicated time points. (D–F) A431 cells were treated with esiRNA against Rabankyrin-5 or control treated for 4 d. (D) Western blot analysis revealed a 50% reduction of Rabankyrin-5 in whole-cell lysate. (E and F) Serum-starved cells (1 h) were stimulated with 50 ng/ml EGF in complete medium for the indicated time points in the presence of 5 mg/ml HRP and 2 μg/ml biotinylated transferrin. Values shown are means ± standard deviation and were performed in duplicates. The results are representatives of at least two independent experiments.

Whereas the aforementioned results demonstrate a general requirement for Rab5 and Rabankyrin-5 in pinocytosis, they do not discriminate between clathrin-dependent and-independent endocytosis. To distinguish between the two pathways, we compared the effects of Rabankyrin-5 overexpression on transferrin and horseradish peroxidase (HRP) uptake in NIH3T3 cells, biochemically. A recombinant adenovirus was generated to overexpress Rabankyrin-5 in these cells. In comparison with mock-infected cells, Rabankyrin-5 overexpression increased the uptake of HRP, particularly after longer (10 min) incubation times (Figure 7A). In contrast, the rate of transferrin uptake was unaffected, suggesting that the increase of fluid phase was not due to increased clathrin-mediated endocytosis (Figure 7C). The increased intracellular accumulation of HRP induced by Rabankyrin-5 seems to be due to increased uptake rather than inhibited recycling, as the latter process was only slightly decreased (Figure 7B).

To determine whether Rabankyrin-5 is also required for macropinocytosis, we attempted to ablate the protein by RNA interference using endoribonuclease-prepared small interfering RNA (esiRNA) (Yang et al. 2002). Since we optimised this technique in human cells, we conducted these experiments in EGF-stimulated A431 cells where an approximately 55% knock-down of Rabankyrin-5 was achieved (Figure 7D). EGF stimulation of control-treated cells enhanced HRP uptake 2-fold versus control-treated, non-EGF-stimulated cells, as reported previously. Reduction of Rabankyrin-5 expression in esiRNA-transfected cells caused an inhibition of HRP of up to approximately 50% by selectively affecting the burst of pinocytosis induced by EGF (within 5 min) (Figure 7E). Under the same conditions, transferrin uptake was marginally inhibited (Figure 7F). We conclude from these experiments that inhibition of fluid-phase uptake occurs due to an inhibition of (macro)pinocytosis rather than of clathrin-mediated endocytosis.

To confirm the requirement of Rabankyrin-5 for fluid-phase endocytosis we used an independent method: quantification of the uptake of fluorescent dextran in NIH3T3 cells microinjected with affinity-purified anti–Rabankyrin-5 antibodies. Following microinjection, FITC-dextran uptake was internalised for 30 min to label macropinosomes. Microinjection of anti–Rabankyrin-5 antibodies inhibited FITC-dextran uptake by 53% ± 5% when compared with cells injected with preimmune serum (Figure S2), whereas the uptake of rhodamine-transferrin was not significantly changed. Altogether, these results suggest that Rabankyrin-5 is a Rab5 effector required for the formation of macropinosomes.

### Rabankyrin-5 Regulates Apical Fluid-Phase Endocytosis in Polarised Epithelial Cells

**Figure 8:**
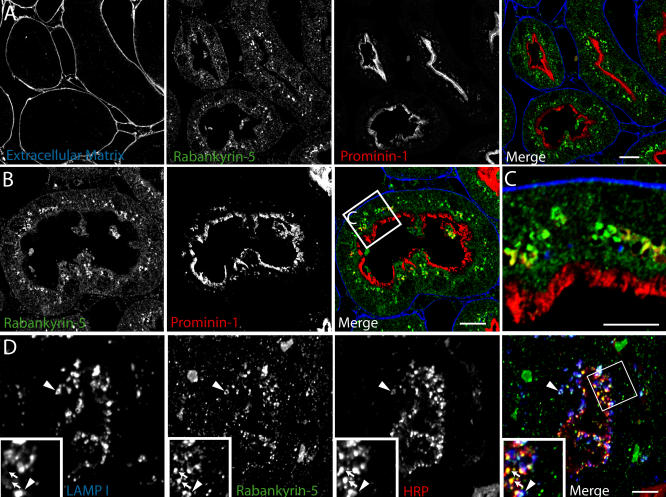
Rabankyrin-5 Localises on Vesicular Structures Underlying the Apical Brush Border in Mouse Kidney Proximal Tubules Mice were perfused either (A–C) directly with 4% PFA or (D) 5 min after injection of HRP into the femoral vein. Immunofluorescence was performed on semi-thin cryosections (500 nm). (C) Higher magnification of the inset in (B). Scale bars represent 10 μm.

Apical pinocytosis in polarised epithelial cells and growth factor-induced macropinocytosis share mechanistic properties, like dependence on actin (Gottlieb et al. 1993) and PI3-K activity (Tuma et al. 1999; Sandvig et al. 2000). In view of the role of Rab5 in endocytic trafficking of polarised cells such as epithelial cells and neurons (Bucci et al. 1994; de Hoop et al. 1994), we explored the role of Rabankyrin-5 in apical endocytosis in polarised epithelial cells. First, we determined the intracellular localisation of Rabankyrin-5 on cryosections of adult mouse kidney, since the epithelial cells in this organ, especially proximal tubule cells (Christensen et al. 1998), exhibit high levels of apical pinocytosis under physiological conditions. Apical endocytic vesicles were pulse-labelled by injecting mice with HRP followed by fixation of the kidney through perfusion after 5 min. Prominin-1 was used as marker for the apical brush border of proximal tubules (Weigmann et al. 1997) in combination with affinity-purified anti–Rabankyrin-5 antibodies. Basement membrane staining was demonstrated by immunolabelling of endogenous mouse immunoglobulin G (IgG). As illustrated in Figure 8, Rabankyrin-5 immunoreactivity was mainly detected on vesicle-like structures underneath the apical, prominin-1–labelled brush border. Most of the vacuole-like Rabankyrin-5 structures contained HRP, suggesting that they correspond to apical endocytic structures (Figure 8D, arrows). By further investigating the nature of these Rabankyrin-5– and HRP-labelled structures, we observed that some vesicles were immunoreactive for LAMP-1 (Figure 8D, arrowheads), suggesting that Rabankyrin-5 (macro)pinocytic vesicles can acquire characteristics of late endocytic compartments.

**Figure 9:**
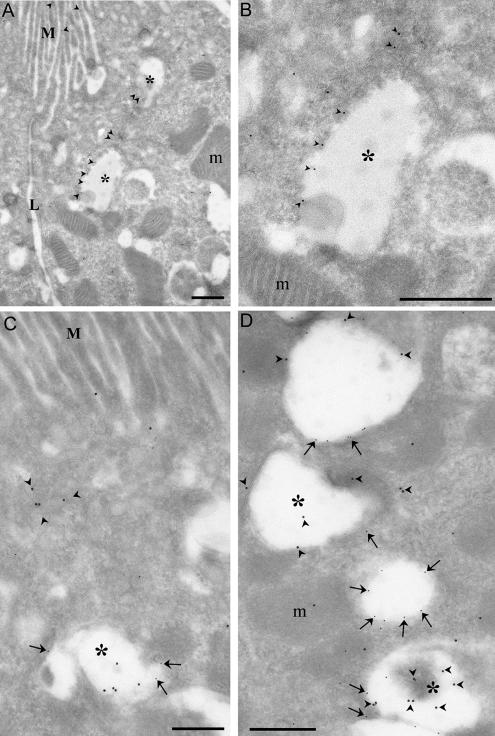
Immunolocalisation of Rabankyrin-5 in the Mouse Kidney Mouse kidney cortex was processed for frozen section immunoelectron microscopy. Sections were (A and B) single labelled for Rabankyrin-5 (arrowheads, 10 nm) or (C and D) double labelled (arrows, 5 nm) for Rabankyrin-5 and LAMP-1. (A) Low-magnification view of the apical region of two proximal tubule cells demonstrates low labelling for Rabankyrin-5 on apical microvilli (M) but stronger labelling (arrowheads) of large subapical electron-lucent vesicular structures (asterisks). One of these structures is shown at higher magnification in (B). L, lateral membrane. (C) Rabankyrin-5 labels LAMP-1–negative subapical structures as well as compartments showing low LAMP-1 labelling (arrows and asterisk). (D) Rabankyrin-5 (arrowheads) associates with compartments, which show no or weak labelling for LAMP-1 (asterisks). In addition, low Rabankyrin-5 labelling is associated with more strongly labelled LAMP-1–positive compartments. Note that there is some nonspecific labelling of mitochondria (m). Scale bars represent 500 nm.

This was confirmed and extended by immunogold electron microscopy on ultrathin frozen sections of mouse proximal tubule. Rabankyrin-5 showed weak labelling of the apical microvilli-covered surface of proximal tubule cells but labelled large electron-lucent structures underlying the apical surface (Figure 9A and 9B). Consistent with the immunofluorescence analysis, double labelling with LAMP-1 antibodies confirmed that many of these structures were LAMP-1 negative or weakly labelled. However, a significant number of Rabankyrin-5–positive structures were also LAMP-1 positive. Rabankyrin-5 immunoreactivity was also observed in distal tubule cells and other segments of the nephron.

**Figure 10:**
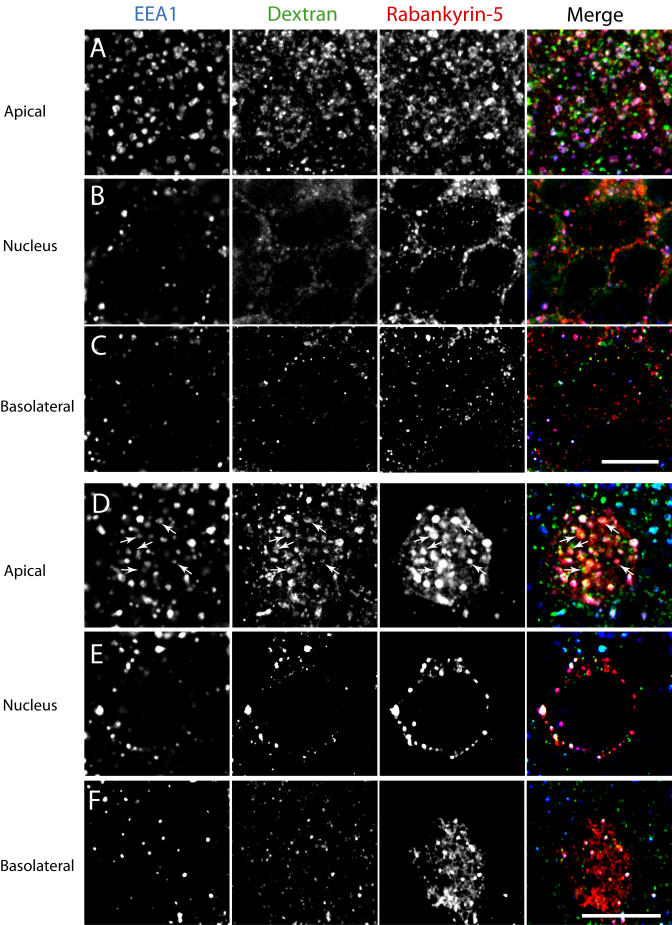
Rabankyrin-5 Colocalises to Apically Labelled Structures in Filter-Grown MDCK Cells MDCK cells were seeded on polycarbonate filters for 4 d and either (A–C) mock-infected or (D–F) infected with an adenovirus construct expressing full-length Rabankyrin-5 for another 18 h. Cells were then incubated for 13 min with 5 mg/ml Alexa 488 dextran, added to the apical medium, fixed, and stained for the indicated antigens. Images represent confocal scans just beneath (A and D) the apical plasma membrane, (B and E) the nuclear area, and (C and F) the basal plasma membrane. Endogenous Rabankyrin-5 and EEA1 localise to several dextran-labelled structures, often with ring-shaped appearance in the apical region. Overexpression of Rabankyrin-5 seems to increase the number of enlarged and dextran-labelled structures, some of which are depleted of EEA1 (arrows) (B). Scale bars represent 10 μm.

**Figure 11:**
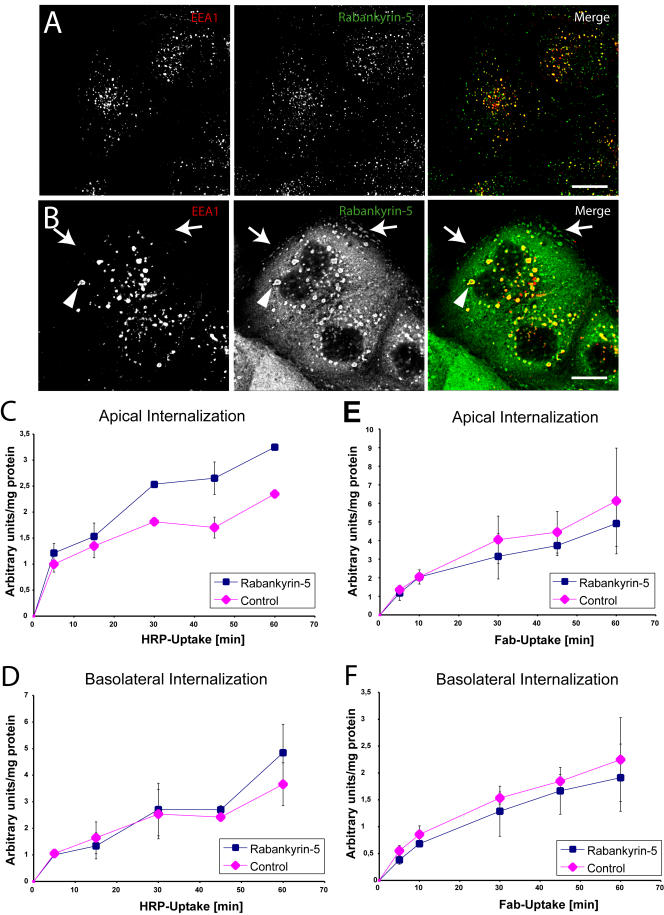
Overexpression of Rabankyrin-5 Stimulates Fluid-Phase Uptake from the Apical Plasma Membrane (A and B) MDCK cells plated on coverslips were (A) mock-infected or (B) infected with Rabankyrin-5 and processed for immunofluorescence. Under both conditions EEA1 colocalised almost completely with Rabankyrin-5, while there were some Rabankyrin-5 structures devoid of EEA1. Upon Rabankyrin-5 overexpression these EEA1-lacking structures were enlarged macropinocytic-like vesicles localised predominantly in the periphery of the cell, while other enlarged compartments containing EEA1 were centripetally located. (C–F) Filter-grown MDCK cells were either mock-infected or infected with recombinant adenovirus for full-length Rabankyrin-5. HRP (5 mg/ml) or biotinylated Fab fragments (50 μg/ml) were internalised from the indicated chambers for various time points. Whereas Rabankyrin-5 overexpression (C) specifically increased HRP uptake from the apical domain, (D) basolateral HRP uptake and (E and F) Fab uptake at both domains were unaffected (*p* < 0,01). Values shown are means ± standard deviation of at least three independent experiments. Scale bars represent 10 μm.

To functionally address the role of Rabankyrin-5 in pinocytosis, we used filter-grown Madin-Darby canine kidney (MDCK) cells as an established system to study polarised trafficking. Alexa 488 dextran was internalised for 13 min from either the apical or the basolateral chamber. Although endogenous Rabankyrin-5 exhibited partial colocalisation with fluid-phase marker internalised from the basolateral side (unpublished data), a striking degree of overlap between the internalised tracer and Rabankyrin-5 on often ring-shaped structures underlying the apical region was observed (Figure 10A). These large vesicles were neither detectable in the nuclear area (Figure 10B) nor close to the basolateral domain (Figure 10C). We next tested whether Rabankyrin-5 overexpression could stimulate fluid-phase uptake from the apical plasma membrane domain. Rabankyrin-5 was overexpressed using the recombinant adenovirus system in MDCK cells grown on coverslips and the cells stained for Rabankyrin-5 and EEA1. As in A431 and NIH3T3 cells, and similar to endogenous Rabankyrin-5 in MDCK cells (Figure 11A), the overexpressed protein exhibited significant colocalisation with endogenous EEA1, but in addition, it induced the formation of enlarged structures devoid of EEA1 in the periphery of the cell (Figure 11B). The recombinant adenovirus was next applied to infect filter-grown MDCK cells. In these cells, it induced enlarged Rabankyrin-5 structures predominantly underlying the apical domain that contained dextran internalised from the apical side. Some of these structures were EEA1 positive, whereas others lacked this marker of early endosomes (see Figure 10D, arrows). These structures were also detectable to some extent in the nuclear region (see Figure 10E) and basolateral membrane domain (see Figure 10F). In addition, there were some enlarged Rabankyrin-5 vesicles that were labelled for neither dextran nor EEA1. These may correspond to macropinosomes that were formed prior to the labelling with the fluorescent fluid-phase marker.

We next measured fluid-phase uptake biochemically by quantifying the internalisation of HRP either from the apical or the basolateral side for various periods of time. Filter-grown MDCK cells were either infected with adenovirus encoding Rabankyrin-5 or control virus prior to HRP internalisation. While internalisation of HRP from the basolateral plasma membrane did not significantly vary (Figure 11D), uptake from the apical membrane was increased up to 65% in cells overexpressing Rabankyrin-5 when compared with control cells (Figure 11C). Given that Rabankyrin-5 was overexpressed in only 50–60% of the cells, this value may be an underestimate. Since the kinetic profiles resembled the ones obtained in NIH3T3 cells, we also tested whether the increase in HRP uptake may have been due to an inhibition of recycling or transcytosis. Neither of these trafficking pathways was significantly affected (unpublished data). To verify that the increase in fluid-phase uptake was not due to stimulation of clathrin-mediated endocytosis, we measured the internalisation of FcLR 5–27, a chimeric receptor between the IgG Fc receptor and the LDL receptor which, when expressed in MDCK cells, is targeted to, and internalised from, both apical and basolateral plasma membrane domains (Matter et al. 1992). Rabankyrin-5 overexpression did not affect significantly the internalisation of FcLR 5–27, as revealed by the uptake of a biotinylated Fab-fragment of the 2.4G2 monoclonal anti-FcRII antibody (Figure 11E and 11F). Taken together, these findings indicate that Rabankyrin-5 is predominantly localised to apical endocytic structures and specifically stimulates apical, non-clathrin-mediated fluid-phase endocytosis upon overexpression in polarised epithelial cells.

## Discussion

### Rabankyrin-5 in Macropinocytosis

Since the pioneering work of Bar-Sagi and Feramisco (1986) almost two decades ago, it has been established that certain oncogenes and signalling molecules induce actin-dependent membrane ruffling and macropinocytosis. Subsequent work has advanced our understanding of the signalling pathway that led to growth factor-dependent actin remodelling and membrane ruffling (Ridley 1994). Despite macropinocytosis being the most ancient form of pinocytosis and an endocytic process of high physiological importance (Amyere et al. 2002), progress in determining the molecular mechanisms that regulate the generation and trafficking of macropinosomes has advanced to a lesser extent. It has been demonstrated that macropinocytosis differs from clathrin-mediated endocytosis (West et al. 1989) and that the two pathways lead to distinct endocytic structures (Hewlett et al. 1994). Rab5, an established regulator of clathrin-mediated endocytosis and endosome dynamics (Gorvel et al. 1991; Bucci et al. 1992; McLauchlan et al. 1998), has also been implicated in macropinocytosis (Li et al. 1997). This hypothesis was based on the expression of mutants that induced or inhibited fluid-phase endocytosis and formation of giant endocytic vesicles. In those studies, however, an accurate assessment of whether the enlarged compartments corresponded to macropinosomes or were produced by the coalescence of multiple endosomes by homotypic fusion was not determined.

Here, we provide evidence that Rabankyrin-5 is a novel Rab5 effector which, in addition to being localised to early endosomes, is associated with macropinosomes and promotes their formation according to previously established criteria. First, Rabankyrin-5 localised to enlarged vesicles that were stimulated by growth factors (e.g., EGF). Second, these vesicles differed from early endosomes by the lack of constitutively endocytosed molecules, such as transferrin, and components of the transport machinery, such as EEA1. Third, the vacuoles originated from the plasma membrane and engulfed extracellular fluid. Fourth, their formation depended on PI3-K activity, and fifth, they followed actin rearrangements at the plasma membrane.

### Possible Functions of Rabankyrin-5

Macropinosomes are formed by the closure of membrane protrusions generated upon actin-mediated membrane ruffling. Although membrane ruffling is required for macropinocytosis, it seems not to be sufficient for macropinosome formation (Araki et al. 1996; West et al. 2000). Our data suggest that Rabankyrin-5 is a novel regulator of this endocytic mechanism. One possibility is that it could play an active role in the generation of macropinosomes (see Figure 7). The deduced primary structure of Rabankyrin-5 predicts the existence of several protein-protein interaction motifs, suggesting a role as a multifunctional adaptor protein. The N terminus of Rabankyrin-5 contains a BTB/POZ motif, which is present in proteins involved in signalling, development, and tumorigenesis and mediates homo- and heterodimerisation (Bardwell and Treisman 1994; Kobayashi et al. 2000). It contains 21 ANK repeats which, by analogy with the function of ANKs (Bennett and Chen 2001; Denker and Barber 2002a), could interact with different proteins on the membrane, including Rab5, and mediate the assembly of a multiprotein complex. Such a scaffolding function has been postulated to bridge the membrane to the actin cytoskeleton but also to link proteins involved in endocytosis and signal transduction (Pryciak and Hartwell 1996; Lubman et al. 2004). Rabankyrin-5 could assemble a membrane-cytoskeleton scaffold committing the ruffling membrane to generate a pinocytic vesicle or directly participate in the closure of the plasma membrane sealing the vesicle. Alternatively, Rabankyrin-5 could be recruited onto macropinosomes concomitantly or following their formation to prevent their back-fusion with the plasma membrane or regulate their onward trafficking. This possibility receives support from our time-lapse video microscopy analysis, where the dissociation of Rabankyrin-5 from the membrane precedes what appears to be the regurgitation of macropinosomes back to the plasma membrane (as shown in Video S1). Interestingly, it has recently been proposed that ANK repeats may not be simply anchoring domains but may be part of a mechanical sensory system that transmits tension from the cytoskeleton to ion channels in the membrane (Corey and Sotomayor 2004; Howard and Bechstedt 2004). In the case of Rabankyrin-5, the ANK repeats may confer a sensory function to detect and adapt to rearrangements of the actin cytoskeleton during membrane ruffling and membrane closure. Interactions of the ANK repeats with ion channels may sense changes in water-salt homeostasis and pH, and respond to them via modulation of macropinocytosis and macropinosome trafficking (de Baey and Lanzavecchia 2000; Morris and Homann 2001; Denker and Barber 2002b).

### Functional Relation between Macropinosomes and Endosomes

How does the function of Rab5 and Rabankyrin-5 in macropinocytosis relate to early endosome trafficking? We have previously proposed a model whereby Rab5, via the recruitment of its effectors, generates and maintains a spatially restricted and functionally specialised membrane domain on the early endosomes (Zerial and McBride 2001). The Rab5 effector hVps34 (Christoforidis et al. 1999b) is a PI3-K that generates PI(3)P. Together with Rab5, this phosphoinositide serves as a binding determinant for the FYVE-finger Rab5 effectors EEA1 (Simonsen et al. 1998) and Rabenosyn-5 on the early-endosome membrane (Nielsen et al. 2000). The Rab5 effectors form oligomeric complexes (McBride et al. 1999) and play distinct but cooperative roles in membrane tethering, fusion, and motility of early endosomes along microtubules (Nielsen et al. 1999). Our data extend the list of PI(3)P-binding Rab5 effectors to Rabankyrin-5. Rabankyrin-5 colocalises with Rab5 and EEA1 to early endosomes and plays a role in homotypic early-endosome fusion, further underscoring the activity of Rab5 as organiser of an endosomal Rab5 domain enriched in PI(3)P-binding effector proteins. Rabankyrin-5 played a minor modulatory role in the fusion of CCVs with early endosomes, suggesting mechanistic differences between homotypic early-endosome fusion and the heterotypic fusion of CCVs with early endosomes. Clearly, Rabankyrin-5 exerted the most striking effects on fluid-phase rather than clathrin-mediated endocytosis.

Clathrin-dependent endocytosis and macropinocytosis are independent but interconnected pathways. Whereas macropinosomes and early endosomes remain segregated in EGF-stimulated A431 cells (Hewlett et al. 1994), they can fuse in other cells, such as dendritic cells and macrophages (Racoosin and Swanson 1993). Rabankyrin-5 alone may not be sufficient for macropinosomes to fuse with endosomes, and this activity may require the additional recruitment of EEA1 and Rabenosyn-5. The extent to which macropinosomes can recruit Rabankyrin-5, release or retain it, and further acquire late endocytic components or other Rab5 effectors acting in endosome tethering and fusion may depend on the cellular context. In kidney cells, for example, several Rabankyrin-5–positive structures appeared also to contain EEA1 and late endocytic markers (LAMP-1), suggesting that macropinosomes can communicate with other endocytic organelles and undergo maturation as it has been described for phagocytosis (Allen and Aderem 1996). Also intriguing is the weak interaction of Rabankyrin-5 with PI(5)P (see Figure 1E) and recent findings that BTB domain-containing proteins interact with ubiquitin ligases (Furukawa et al. 2003). Since both components have been implicated in the biogenesis of multivesicular bodies (Katzmann et al. 2001), Rabankyrin-5 may function in the trafficking to late endocytic components, as it was described previously for PIKfyve, another FYVE-finger–containing protein (Ikonomov et al. 2003).

Stimulation of epithelial cells with either EGF or phorbol esters increases fluid-phase uptake while reducing internalisation via clathrin-mediated endocytosis (Sandvig and van Deurs 1990). Our results provide a possible explanation for how the two endocytic routes can be quantitatively balanced. Since Rab5 appears to be rate limiting for both receptor-mediated and fluid-phase endocytosis, its activity could be shifted between these two endocytic pathways, depending on the stimuli and the cell type. The shared activity of Rab5 and Rabankyrin-5 would ensure coordination between endosome trafficking and macropinocytosis, regulate the kinetics, and limit the extent of both endocytic processes, thus preserving plasma membrane homeostasis.

### Rabankyrin-5 in Apical, Actin-Dependent Endocytosis in Polarised Epithelial Cells

In addition to macropinocytosis in nonpolarised cells, we have found that Rabankyrin-5 regulates apical, non-clathrin-mediated pinocytosis in polarised epithelial cells. The role of apically stimulated pinocytosis is crucial for the physiology of various organs. For example, it plays an important role in the reabsorption of proteins from the glomerular filtrate in the renal proximal tubule (Christensen et al. 1998). Rabankyrin-5 may be a regulator of this process and this possibility is supported by our findings, which show that it (1) localises to subapical compartments in kidney proximal tubules and (2) induces fluid-phase, clathrin-independent uptake from the apical but not basolateral side in polarised MDCK cells. As for macropinocytosis, apical endocytosis depends on actin remodelling mediated by Rho family GTPases (Gottlieb et al. 1993; West et al. 2000) and ARF6 (Altschuler et al. 1999). Since macropinocytosis appears to occur primarily from the apical side, the involvement of epidermal growth factor receptor as demonstrated for nonpolarised cells (Haigler et al. 1979; West et al. 1989) is unlikely since it is primarily localised to the basolateral domain of polarised epithelial cells (Gesualdo et al. 1996). However, apical pinocytosis depends on other components of receptor tyrosine kinase signalling pathways such as PI3-K (Tuma et al. 1999) and protein kinase C and can be induced by oncogenes (e.g., v-Src) that stimulate macropinocytosis in nonpolarised cells (Holm et al. 1995; Amyere et al. 2002). It is not known which PI3-K subtype functions in apical pinocytosis, but an attractive candidate is PI3-Kβ, as it has been identified as a Rab5 effector (Christoforidis et al. 1999b). Its activity could be regulated by Rab5 constitutively and/or be subjected to stimulation by apical or axonal signalling molecules (Pillion et al. 1989; Kryl et al. 1999). In neurons, Rac- and actin-dependent endocytosis of Eph receptor-ephrin complexes is required to control repulsive versus attractive cell movement during tissue patterning in embryonic development (Marston et al. 2003; Zimmer et al. 2003) and may play a role in the structural plasticity of synapses (Holt et al. 2003). It will be interesting to determine whether Rabankyrin-5 and the macropinocytic machinery play a role in this event.

In conclusion, the identification of Rabankyrin-5 opens new opportunities for investigating the molecular principles underlying macropinocytosis and its regulation by signalling molecules. The dual role of Rab5 and Rabankyrin-5 in endosome and macropinosome function argues for a role in coordinating these two different endocytic mechanisms. Future work is required to identify the molecular partners of Rabankyrin-5 and to establish the mechanisms responsible for its membrane targeting and its role in endocytic membrane dynamics.

## Materials and Methods

### 

#### Reagents and cell lines.

Phospholipids were purchased from Sigma (St. Louis, Missouri, United States) and Calbiochem (San Diego, California, United States). FITC- or Texas Red-conjugated dextran (lysine fixable; MW, 10.000 and 70.000), rhodamine-conjugated EGF, and rhodamine-conjugated transferrin were purchased from Molecular Probes (Eugene, Oregon, United States). Wortmannin and LY 294002 were from Calbiochem. A431 and NIH3T3 cells were grown in DMEM (high glucose) supplemented with 10% (v/v) heat-inactivated foetal calf serum, 100 U/ml penicillin, 100 μg/ml streptomycin, and 2 mM L-glutamine. MDCK II cells were grown in MEM plus 10% FCS. MDCK II cells stably transfected for FcLR 5–27 (Matter et al. 1992) were a gift of Professor Ira Mellman.

#### Amino acid sequence determination and Rabankyrin-5 cloning

The 130-kDa protein band was excised from gels and enzymatically digested. The tandem mass spectroscopy protein sequencing procedure was performed as described previously (Wilm et al. 1996). Bovine peptides determined from Rabankyrin-5 were from bovine brain and were used to identify corresponding expressed sequence tags using BLAST similarity searches at NCBI. A random primed HeLa cDNA library was used in a PCR reaction with primers based on the mouse cDNA to obtain the full-length clone of Rabankyrin-5.

#### Preparation of recombinant Rabankyrin-5

Recombinant proteins of Rabankyrin-5 were expressed in SF plus insect cells according to the manufacturer's instructions using Rabankyrin-5 cDNA subcloned into pfastBAC vector or subcloned into pGEX-6P series and expressed in BL21 cells.

#### Antibodies, plasmids, and recombinant adenovirus

Human anti-EEA1 serum (1:10.000) was a gift from Ban Hok Toh (Monash Medical School, Adelaide, Australia), whereas a mouse monoclonal EEA1 antibody, a sheep polyclonal anti-HRP antibody and an antibody against phosphotyrosine (α-4G10) were purchased from BD Bioscience (Heidelberg, Germany), Abam (Cambridge, United Kingdom), and Upstate Biotechnology (Lake Placid, New York, United States), respectively. Fluorescently labelled secondary antibodies were purchased from Molecular Probes. Recombinant full-length Rabankyrin-5 was used to raise a polyclonal antibody in rabbit. Human full-length Rabankyrin-5 was cloned into pEYFP vectors containing an N-terminal tag (pEYFP-C1; Clontech, Palo Alto, California, United States). Recombinant adenovirus encoding full-length Rabankyrin-5 was generated according to the manufacturer's protocol (AdEasy). cDNAs encoding Rab5wt were fused to the amino termini of ECFP or EYFP, as previously described (Sonnichsen et al. 2000). The antibody for the FcLR 5-27 chimeric receptor was purified from 2.4G2 hybridoma supernatant (Unkeless 1979) by ammonium sulfate precipitation. For the preparation and biotinylation of Fab fragments of 2.4G2, 6 mg of purified 2.4G2 IgG was digested with insoluble papain enzyme as described by the manufacturer (Sigma). Fab fragments were biotinylated with NHS-LC-biotin or NHS-SS-biotin (Pierce, Rockford, Illinois, United States).

#### In vitro binding assays

The GST-Rab5 affinity chromatography was performed as in Christoforidis and Zerial (2000). ^35^S-Methionine–labelled proteins were transcribed and translated in vitro using a TnT-coupled transcription-translation kit (Promega, Madison, Wisconsin, United States). For Rab5 effector recruitment assays, in vitro-translated proteins were incubated with glutathione-sepharose beads complexed with GST-Rab5-GTPγS or GST-Rab5-GDP and eluted as described previously (Christoforidis et al. 1999a).

#### Confocal immunofluorescence microscopy and transfection

Cells were transfected with plasmids using FuGENE6 according to the manufacturer's instructions (Roche, Basel, Switzerland). After 16–24 h, transfected cells were washed twice with PBS and fixed with 3% paraformaldehyde and immunofluorescence labelling was performed according to standard procedures. Cells were mounted in moviol and examined on a confocal microscope (Leica TCS SP2; Leica, Wetzlar, Germany) with a 100×/1.40 plan-Apochromat objective. Fluorescent images were collected using the Leica IM500 Image Manager and processed using Adobe Photoshop v5.5 (Adobe Systems, San Jose, California, United States).

#### Endocytosis of FITC-labelled dextran and rhodamine-labelled transferrin, and quantification

NIH3T3 cells grown on glass coverslips and transfected with plasmids encoding RN-tre were incubated with 3 mg/ml FITC-labelled dextran (MW, 70.000) in DMEM supplemented with 1% FCS and 20 mM HEPES (pH 7,4), fixed, and stained with rhodamine-conjugated antirabbit secondary antibody. Dextran uptake was determined by quantifying grey values of thresholded, fluorescent images of at least 15 cells using MetaVue 6.1r3 and ImageJ (NIH).

#### In vitro endosome fusion and early endosome/liposome recruitment assays

In vitro fusion assays were performed using early endosomes labelled with biotinylated transferrin or antitransferrin antibody as well as CCVs labelled with biotinylated transferrin prepared from HeLa cells as described previously (Horiuchi et al. 1997). The concentration of Rabankyrin-5 in HeLa cell cytosol in the in vitro early-endosome fusion assay was estimated to be approximately 50 nM, as determined by comparison with a serial dilution of recombinant Rabankyrin-5 analysed by SDS-PAGE, followed by Western blotting analysis. Recruitment of cytosolic proteins to early endosomes was performed as described in Christoforidis et al. (1999b). Recombinant Rabankyrin-5 was recruited to phosphoinositides as described previously (Nielsen et al. 2000) using liposomes (98% phosphatidylcholine, 2% phosphoinositides; 2 mg/ml final concentration in 30 mM HEPES-NaOH [pH 7,2], 120 mM NaCl, 0,5 mM EGTA) prepared as reported previously (Otter-Nilsson et al. 1999).

#### Time-lapse video microscopy and data processing

Time-lapse epifluorescence video microscopy was performed using an Olympus IX70 (Olympus, Hamburg, Germany) inverted microscope equipped with a polychrome II monochromator (TILL Photonics, Martinsried, Germany), a custom filter block for simultaneous visualization of YFP and CFP (AHF Analysentechnik, Tübingen, Germany), a 100× oil-immersion objective (NA 1.35, Olympus) attached to a PIFOC z-SCAN (Physik Instrumente, Waldbronn, Germany), an incubation chamber (37 °C), and a 12-bit CCD digital camera IMAGO (0,134 μm pixel-1; 2 times 2 binning) (TILL Photonics), controlled by TILLvisION v3.3 software (TILL Photonics). Time-lapse video sequences of YFP and CFP images were merged as RGBs using TILLvisION v3.3. They were exported as single TIFF files and either further processed using Adobe Photoshop 5.5 and Illustrator 8.0 or converted into QuickTime movies using ImageJ (NIH).

#### HRP staining of the apical endocytic machinery in mouse proximal tubules

Anaesthetized mice were perfusion fixed with 4% PFA, 100 mM HEPES (pH 7,25), and 0,2% sucrose either directly or 5 min after injection with 5 mg/g body weight HRP (Sigma) into the femoral vein. Kidneys were excised and postfixed 6 h at 4 °C. Some tissue pieces were cryoprotected in 2,3 M sucrose overnight at 4 °C, frozen in liquid nitrogen, and cryosectioned on a Leica UCT (Tokuyasu et al. 1984).

#### Adenovirus infection and continuous uptake measurement with HRP, biotinylated transferrin, and biotinylated Fab (2.4G2).

MDCK cells were grown on 12-mm, 0,4-μm pore Transwell polycarbonate filters (Corning Costar, Cambridge, Massachusetts, United States). Ninety-six hours after seeding, cells were infected from the apical side for 4 h at 37 °C with adenoviruses in 250 μl of complete medium. After changing medium, cells were incubated for 18 h at 37 °C and then used for biochemical assays or microscopy. NIH3T3 and MDCK cells grown on coverslips in 24-well plates were infected in 500 μl of medium. HRP uptake was performed as described previously (Bomsel et al. 1989). In brief, cells were incubated in incubation medium (IM; DMEM, 1% FCS, 24 mM HEPES [pH 7,4]) at 37 °C for 1 h. For internalisation experiments coverslips or Transwell filters (Costar) were incubated with IM containing 5–10 mg/ml HRP, 2 μg/ml biotinylated transferrin, or 5 μg/ml biotinylated Fab (2.4G2). In the case of MDCK cells, HRP and Fab were applied from either the apical or the basolateral side. After internalisation, cells were briefly washed with warm IM followed by three washes with ice-cold PBS supplemented with 1 mM CaCl_2_ and 1 mM MgCl_2_ (PBS^++^) containing 2 mg/ml BSA. Cell surface-bound transferrin was efficiently removed (95%) by three incubations over 10 min in IM (pH 3,4). Extracellular HRP was quenched in the cold by incubating the cells for 20 min with 20 mM MESNA in 100 mM NaCl, 50 mM Tris (pH 8,7), followed by two washes with ice-cold PBS and a further incubation for 10 min with ice-cold 50 mM iodoacetamide in PBS. For recycling experiments, cells were transferred again to 37 °C in fresh medium that was collected at the indicated time points. Thereafter, cells were washed once more with PBS and extracted for 15 min at 4 °C with 300 μl of lysis buffer (1% w/v Triton X-100, 0,1% w/v SDS, 20 mM HEPES [pH 7,4], and 100 U/ml DNase). Total HRP activity present in the cells was determined in duplicates out of at least three independent experiments using the previously described enzymatic method (Steinman et al. 1976) and standardised to the protein content of each well. Fab and transferrin uptake assay was performed as described previously (Zacchi et al. 1998).

#### Knock-down of Rabankyrin-5 in A431 cells by RNA interference

A431 cells were seeded in 24-well plates the day prior to transfection. Cells were transfected using oligofectamine (Invitrogen, Carlsbad, California, United States) with either 300 ng/ml double- or single-stranded (control) esiRNA derived from the I.M.A.G.E. clone (IMAGE:258664) according to the protocol of Yang et al. (2002). After 4 d, cells were starved in MEM containing 2 mg/ml BSA and 24 mM HEPES (pH 7,3) for 1 h and stimulated with 50 ng/ml EGF in complete medium containing 24 mM HEPES (pH 7,3), 5–10 mg/ml HRP, and 2 μg/ml biotinylated transferrin for the indicated time points (see “Adenovirus infection and continuous uptake measurement with HRP, biotinylated transferrin, and biotinylated Fab (2.4G2)” above).

#### Electron microscopy

Cells or tissues were fixed with 8% PFA in PHEM buffer and then processed for frozen sectioning according to published methods (Liou et al. 1996). Thawed sections were either single or double labelled. For single labelling a polyclonal anti–Rabankyrin-5 antibody followed by protein A 10 nm gold (University of Utrecht) was used. For double labelling the polyclonal anti–Rabankyrin-5 antibody was incubated together with rat anti–LAMP-1 (1D4B; courtesy of Professor M. Desjardins, University of Montreal, Montreal, Canada). The sections were then incubated with a mixture of anti-rabbit 10 nm gold and anti-rat 5 nm gold (BBI International, Cardiff, United Kingdom).

## Supporting Information

Figure S1 Wortmannin Treatment Inhibits Fluid-Phase Uptake into Enlarged Rabankyrin-5 Structures NIH3T3 cells were pretreated for 20 min with wortmannin (100 nM) and incubated for 25 min with 0,5 μg/ml rhodamine-labelled transferrin and 2,5 mg/ml FITC-labelled dextran (MW, 70.000), fixed, processed for immunofluorescence, and analysed by confocal scanning microscopy. Scale bar represents 10 μm.
(8.4 MB EPS).

Figure S2 Microinjection of a Function-Blocking Antibody against Rabankyrin-5 Decreases Fluid-Phase Uptake NIH3T3 cells were either microinjected with (A and D) preimmune or (B and C) affinity-purified polyclonal α–Rabankyrin-5 antibodies and subjected to a 30-min incubation at 37 °C with (A and B) 3 mg/ml FITC-conjugated dextran (MW, 70.000) or (C and D) 1 μg/ml rhodamine-conjugated transferrin. Cells injected with immune but not preimmune antibodies showed a significant reduction of the fluid-phase marker, while the uptake of transferrin was not significantly perturbed.
(D) Fluid-phase dextran quantification of single cells microinjected for indicated antibodies by measuring internalised fluorescence intensity (*p* > 0.001). Values shown are means ± standard deviation of at least 15 cells. Scale bars represent 10 μm.
(44.0 MB EPS).

Video S1 Rabankyrin-5 Localizes to Macropinosomes Generated by Actin Dynamics Images were taken every 3 s over a time period of 15 min. RGB stacks were converted to QuickTime format using ImageJ (NIH). The movie is played at 15 frames per second.
(11.1 MB MOV).

### Accession Numbers

The LocusLink (http://www.ncbi.nlm.nih.gov/LocusLink) accession numbers of proteins discussed in this paper are H-Ras (ID 3265), v-Src (ID 6714), Arf6 (ID 382), hVps34 (ID 5289), p110β (ID 5291), Rab34 (ID 83871), Rab5 (ID 5868), Ankhzn (ID 51479), Rab4 (ID 5867), Rab7 (ID 7879), Rab11 (ID 8766), Rabenosyn-5 (ID 64145), EEA1 (ID 8411), Caveolin-1 (ID 857), RN-tre (ID 9712), Transferrin (ID 7018), EGF (ID 1950), LAMP-1 (ID 16783), Prominin-1(ID 8842), PIKfyve (ID 200576), RhoA (ID 387), and PKC-α (ID 5578).

## Abbreviations

ANK: ankyrin
CCV: clathrin-coated vesicle
CFP: cyan fluorescent protein
EGF: epidermal growth factor
esiRNA: endoribonuclease-prepared small interfering RNA
GAP: GTPase-activating protein
GPI: glycosyl-phosphatidylinositol
GST: glutathione S-transferase
GTP: guanine nucleotide trisphosphate
HRP: horseradish peroxidase
IgG: immunoglobulin G
IM: incubation medium
MDCK cells: Madin-Darby canine kidney cells
PI(3)P: phosphatidylinositol-3-phosphate
PI3-K: phosphatidylinositol-3 kinase
PMA: phorbol 12-myristate 13-acetate;YFP
